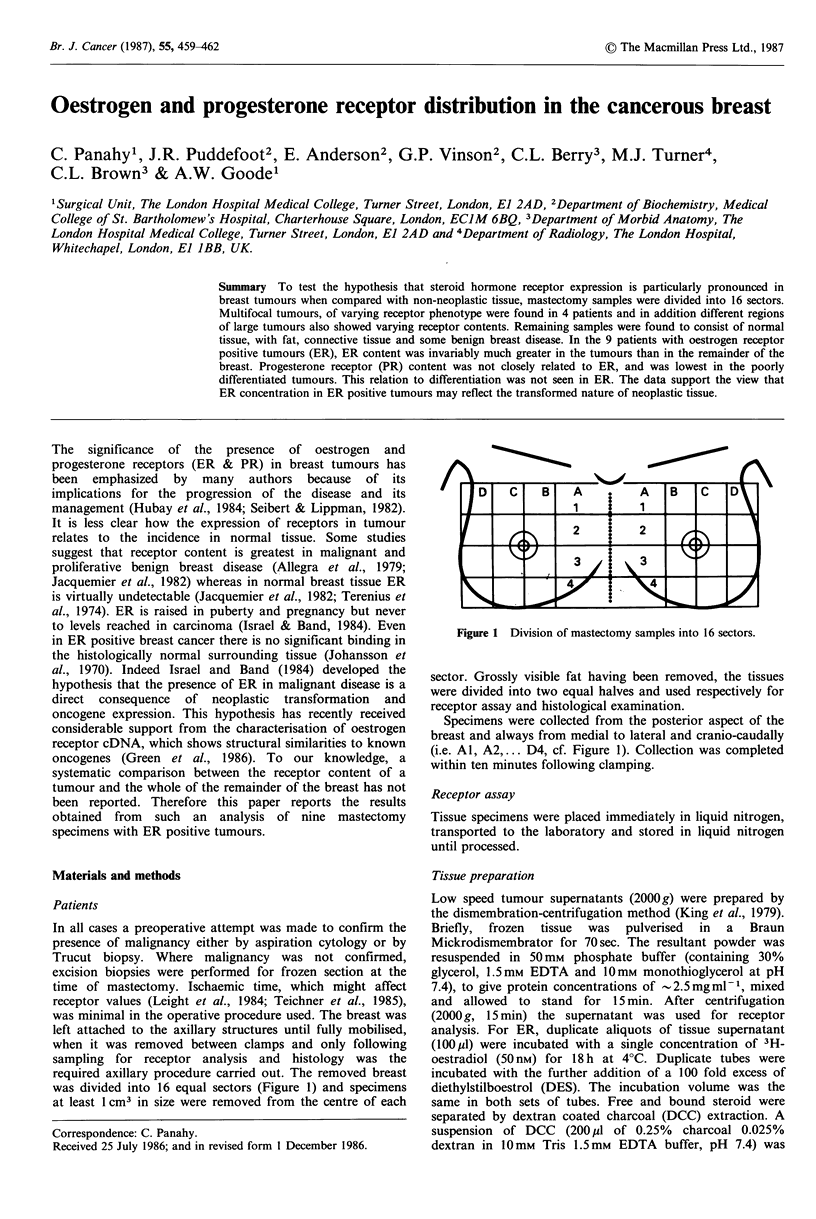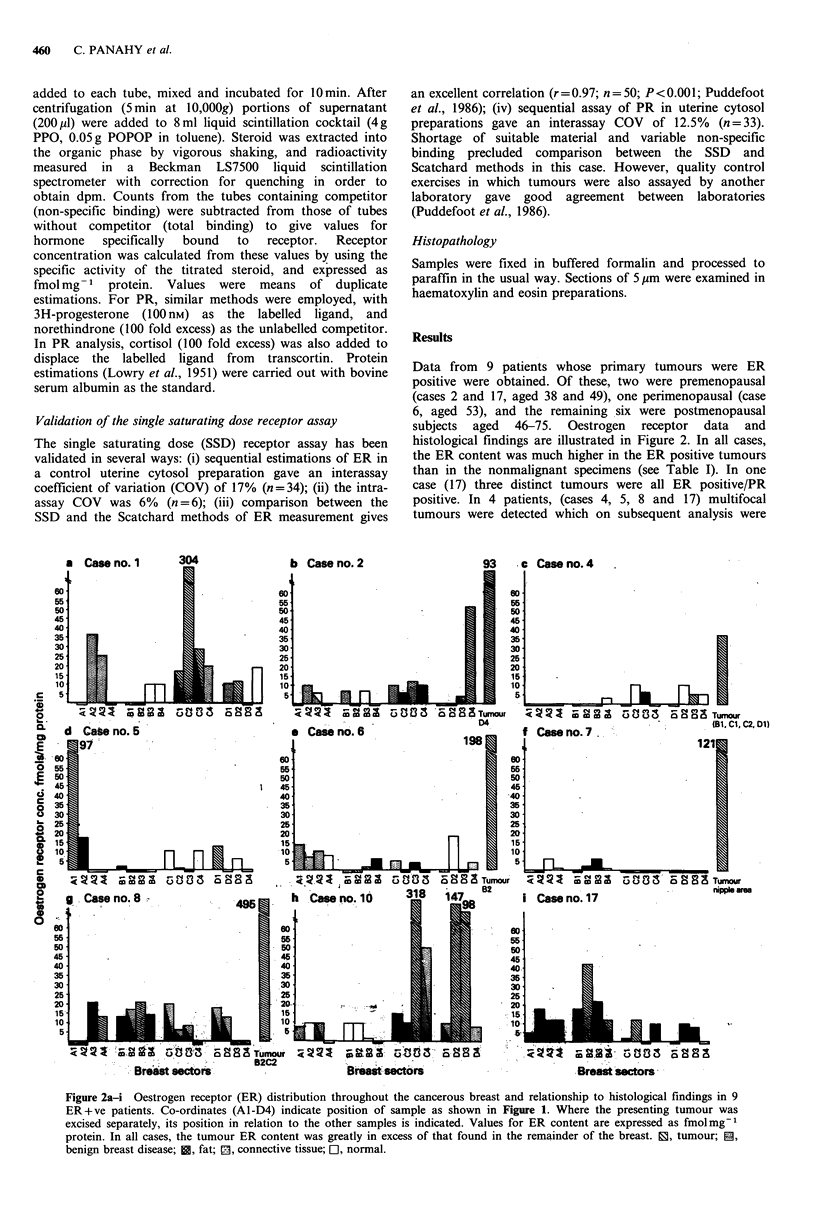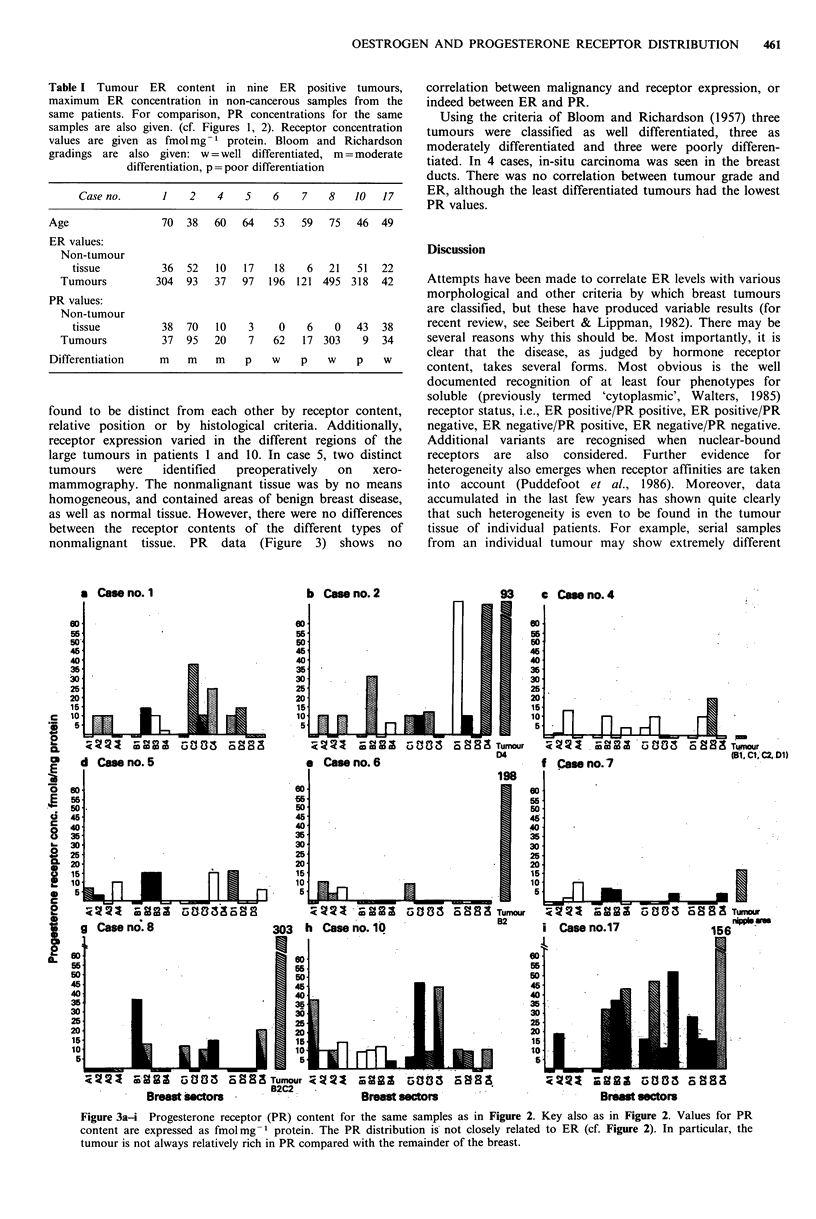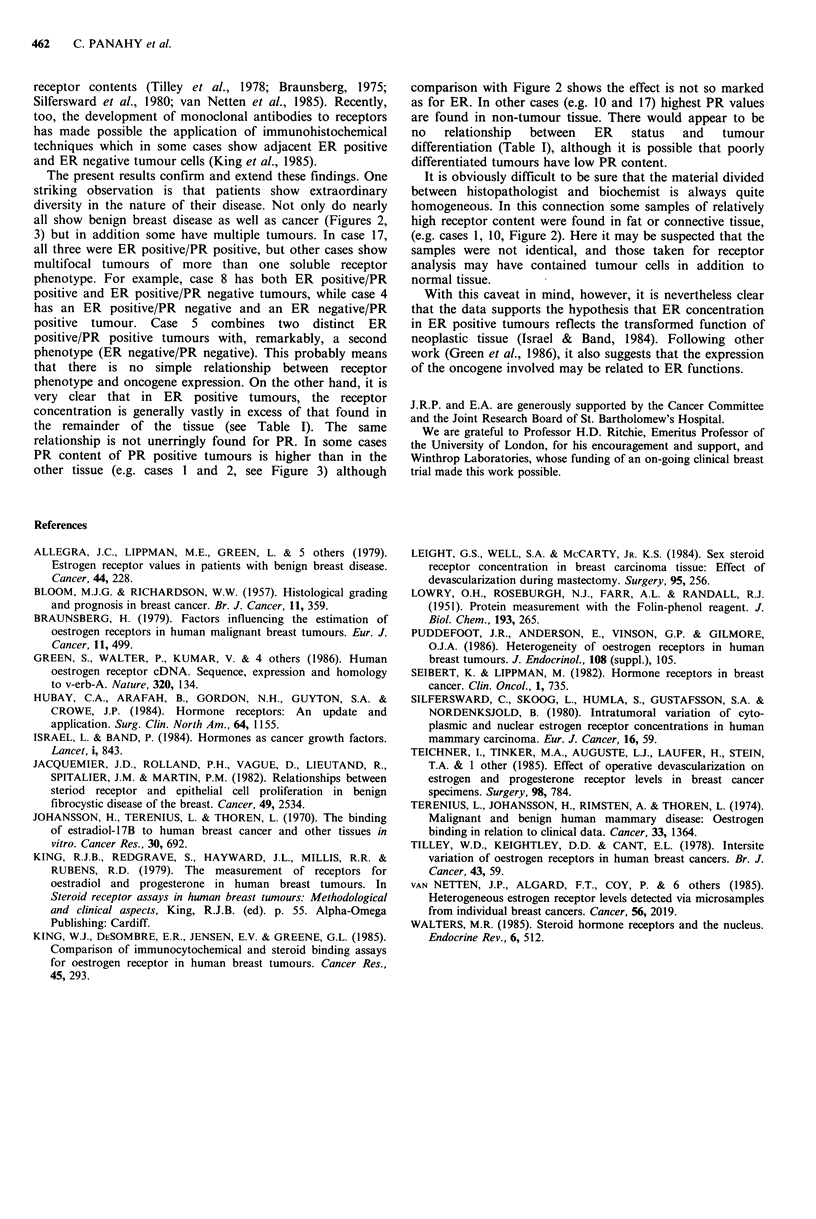# Oestrogen and progesterone receptor distribution in the cancerous breast.

**DOI:** 10.1038/bjc.1987.90

**Published:** 1987-04

**Authors:** C. Panahy, J. R. Puddefoot, E. Anderson, G. P. Vinson, C. L. Berry, M. J. Turner, C. L. Brown, A. W. Goode

## Abstract

To test the hypothesis that steroid hormone receptor expression is particularly pronounced in breast tumours when compared with non-neoplastic tissue, mastectomy samples were divided into 16 sectors. Multifocal tumours, of varying receptor phenotype were found in 4 patients and in addition different regions of large tumours also showed varying receptor contents. Remaining samples were found to consist of normal tissue, with fat, connective tissue and some benign breast disease. In the 9 patients with oestrogen receptor positive tumours (ER), ER content was invariably much greater in the tumours than in the remainder of the breast. Progesterone receptor (PR) content was not closely related to ER, and was lowest in the poorly differentiated tumours. This relation to differentiation was not seen in ER. The data support the view that ER concentration in ER positive tumours may reflect the transformed nature of neoplastic tissue.


*Br. J. Cancer* (1987), **55**, 459–462 © The Macmillan Press Ltd., 1987

### Oestrogen and progesterone receptor distribution in the cancerous breast

C. Panahy[1], J.R. Puddefoot[2], E. Anderson[2], G.P. Vinson[2], C.L. Berry[3], M.J. Turner[4], C.L. Brown[3] & A.W. Goode[1]

[1]*Surgical Unit, The London Hospital Medical College, Turner Street, London, E1 2AD,* [2]*Department of Biochemistry, Medical College of St. Bartholomew's Hospital, Charterhouse Square, London, EC1M 6BQ,* [3]*Department of Morbid Anatomy, The London Hospital Medical College, Turner Street, London, E1 2AD and* [4]*Department of Radiology, The London Hospital, Whitechapel, London, E1 1BB, UK.*

**Summary** To test the hypothesis that steroid hormone receptor expression is particularly pronounced in breast tumours when compared with non-neoplastic tissue, mastectomy samples were divided into 16 sectors. Multifocal tumours, of varying receptor phenotype were found in 4 patients and in addition different regions of large tumours also showed varying receptor contents. Remaining samples were found to consist of normal tissue, with fat, connective tissue and some benign breast disease. In the 9 patients with oestrogen receptor positive tumours (ER), ER content was invariably much greater in the tumours than in the remainder of the breast. Progesterone receptor (PR) content was not closely related to ER, and was lowest in the poorly differentiated tumours. This relation to differentiation was not seen in ER. The data support the view that ER concentration in ER positive tumours may reflect the transformed nature of neoplastic tissue.

The significance of the presence of oestrogen and progesterone receptors (ER & PR) in breast tumours has been emphasized by many authors because of its implications for the progression of the disease and its management (Hubay *et al.*, 1984; Seibert & Lippman, 1982). It is less clear how the expression of receptors in tumour relates to the incidence in normal tissue. Some studies suggest that receptor content is greatest in malignant and proliferative benign breast disease (Allegra *et al.*, 1979; Jacquemier *et al.*, 1982) whereas in normal breast tissue ER is virtually undetectable (Jacquemier *et al.*, 1982; Terenius *et al.*, 1974). ER is raised in puberty and pregnancy but never to levels reached in carcinoma (Israel & Band, 1984). Even in ER positive breast cancer there is no significant binding in the histologically normal surrounding tissue (Johansson *et al.*, 1970). Indeed Israel and Band (1984) developed the hypothesis that the presence of ER in malignant disease is a direct consequence of neoplastic transformation and oncogene expression. This hypothesis has recently received considerable support from the characterisation of oestrogen receptor cDNA, which shows structural similarities to known oncogenes (Green *et al.*, 1986). To our knowledge, a systematic comparison between the receptor content of a tumour and the whole of the remainder of the breast has not been reported. Therefore this paper reports the results obtained from such an analysis of nine mastectomy specimens with ER positive tumours.

### Materials and methods

### *Patients*

In all cases a preoperative attempt was made to confirm the presence of malignancy either by aspiration cytology or by Trucut biopsy. Where malignancy was not confirmed, excision biopsies were performed for frozen section at the time of mastectomy. Ischaemic time, which might affect receptor values (Leight *et al.*, 1984; Teichner *et al.*, 1985), was minimal in the operative procedure used. The breast was left attached to the axillary structures until fully mobilised, when it was removed between clamps and only following sampling for receptor analysis and histology was the required axillary procedure carried out. The removed breast was divided into 16 equal sectors (Figure 1) and specimens at least 1 cm³ in size were removed from the centre of each

**Figure 1** Division of mastectomy samples into 16 sectors.

sector. Grossly visible fat having been removed, the tissues were divided into two equal halves and used respectively for receptor assay and histological examination.

Specimens were collected from the posterior aspect of the breast and always from medial to lateral and cranio-caudally (i.e. A1, A2,... D4, cf. Figure 1). Collection was completed within ten minutes following clamping.

### *Receptor assay*

Tissue specimens were placed immediately in liquid nitrogen, transported to the laboratory and stored in liquid nitrogen until processed.

### *Tissue preparation*

Low speed tumour supernatants (2000 *g*) were prepared by the dismembration-centrifugation method (King *et al.*, 1979). Briefly, frozen tissue was pulverised in a Braun Mickrodismembrator for 70 sec. The resultant powder was resuspended in 50 mM phosphate buffer (containing 30% glycerol, 1.5 mM EDTA and 10 mM monothioglycerol at pH 7.4), to give protein concentrations of ~2.5 mg ml$^{-1}$, mixed and allowed to stand for 15 min. After centrifugation (2000 *g*, 15 min) the supernatant was used for receptor analysis. For ER, duplicate aliquots of tissue supernatant (100 μl) were incubated with a single concentration of $^3$H-oestradiol (50 nM) for 18 h at 4°C. Duplicate tubes were incubated with the further addition of a 100 fold excess of diethylstilboestrol (DES). The incubation volume was the same in both sets of tubes. Free and bound steroid were separated by dextran coated charcoal (DCC) extraction. A suspension of DCC (200 μl of 0.25% charcoal 0.025% dextran in 10 mM Tris 1.5 mM EDTA buffer, pH 7.4) was

Correspondence: C. Panahy.
Received 25 July 1986; and in revised form 1 December 1986.

added to each tube, mixed and incubated for 10 min. After centrifugation (5 min at 10,000g) portions of supernatant (200 μl) were added to 8 ml liquid scintillation cocktail (4 g PPO, 0.05 g POPOP in toluene). Steroid was extracted into the organic phase by vigorous shaking, and radioactivity measured in a Beckman LS7500 liquid scintillation spectrometer with correction for quenching in order to obtain dpm. Counts from the tubes containing competitor (non-specific binding) were subtracted from those of tubes without competitor (total binding) to give values for hormone specifically bound to receptor. Receptor concentration was calculated from these values by using the specific activity of the titrated steroid, and expressed as fmol $mg^{-1}$ protein. Values were means of duplicate estimations. For PR, similar methods were employed, with 3H-progesterone (100 nM) as the labelled ligand, and norethindrone (100 fold excess) as the unlabelled competitor. In PR analysis, cortisol (100 fold excess) was also added to displace the labelled ligand from transcortin. Protein estimations (Lowry et al., 1951) were carried out with bovine serum albumin as the standard.

*Validation of the single saturating dose receptor assay*

The single saturating dose (SSD) receptor assay has been validated in several ways: (i) sequential estimations of ER in a control uterine cytosol preparation gave an interassay coefficient of variation (COV) of 17% ($n=34$); (ii) the intra-assay COV was 6% ($n=6$); (iii) comparison between the SSD and the Scatchard methods of ER measurement gives an excellent correlation ($r=0.97$; $n=50$; $P<0.001$; Puddefoot et al., 1986); (iv) sequential assay of PR in uterine cytosol preparations gave an interassay COV of 12.5% ($n=33$). Shortage of suitable material and variable non-specific binding precluded comparison between the SSD and Scatchard methods in this case. However, quality control exercises in which tumours were also assayed by another laboratory gave good agreement between laboratories (Puddefoot et al., 1986).

*Histopathology*

Samples were fixed in buffered formalin and processed to paraffin in the usual way. Sections of 5 μm were examined in haematoxylin and eosin preparations.

### Results

Data from 9 patients whose primary tumours were ER positive were obtained. Of these, two were premenopausal (cases 2 and 17, aged 38 and 49), one perimenopausal (case 6, aged 53), and the remaining six were postmenopausal subjects aged 46–75. Oestrogen receptor data and histological findings are illustrated in Figure 2. In all cases, the ER content was much higher in the ER positive tumours than in the nonmalignant specimens (see Table I). In one case (17) three distinct tumours were all ER positive/PR positive. In 4 patients, (cases 4, 5, 8 and 17) multifocal tumours were detected which on subsequent analysis were

**Figure 2a–i** Oestrogen receptor (ER) distribution throughout the cancerous breast and relationship to histological findings in 9 ER+ve patients. Co-ordinates (A1-D4) indicate position of sample as shown in **Figure 1**. Where the presenting tumour was excised separately, its position in relation to the other samples is indicated. Values for ER content are expressed as fmol $mg^{-1}$ protein. In all cases, the tumour ER content was greatly in excess of that found in the remainder of the breast. ▧, tumour; ▦, benign breast disease; ▩, fat; ▤, connective tissue; □, normal.

**Table I** Tumour ER content in nine ER positive tumours, maximum ER concentration in non-cancerous samples from the same patients. For comparison, PR concentrations for the same samples are also given. (cf. Figures 1, 2). Receptor concentration values are given as fmol mg$^{-1}$ protein. Bloom and Richardson gradings are also given: w = well differentiated, m = moderate differentiation, p = poor differentiation

| *Case no.* | *1* | *2* | *4* | *5* | *6* | *7* | *8* | *10* | *17* |
|---|---|---|---|---|---|---|---|---|---|
| Age | 70 | 38 | 60 | 64 | 53 | 59 | 75 | 46 | 49 |
| ER values: | | | | | | | | | |
| Non-tumour tissue | 36 | 52 | 10 | 17 | 18 | 6 | 21 | 51 | 22 |
| Tumours | 304 | 93 | 37 | 97 | 196 | 121 | 495 | 318 | 42 |
| PR values: | | | | | | | | | |
| Non-tumour tissue | 38 | 70 | 10 | 3 | 0 | 6 | 0 | 43 | 38 |
| Tumours | 37 | 95 | 20 | 7 | 62 | 17 | 303 | 9 | 34 |
| Differentiation | m | m | m | p | w | p | w | p | w |

found to be distinct from each other by receptor content, relative position or by histological criteria. Additionally, receptor expression varied in the different regions of the large tumours in patients 1 and 10. In case 5, two distinct tumours were identified preoperatively on xeromammography. The nonmalignant tissue was by no means homogeneous, and contained areas of benign breast disease, as well as normal tissue. However, there were no differences between the receptor contents of the different types of nonmalignant tissue. PR data (Figure 3) shows no correlation between malignancy and receptor expression, or indeed between ER and PR.

Using the criteria of Bloom and Richardson (1957) three tumours were classified as well differentiated, three as moderately differentiated and three were poorly differentiated. In 4 cases, in-situ carcinoma was seen in the breast ducts. There was no correlation between tumour grade and ER, although the least differentiated tumours had the lowest PR values.

### Discussion

Attempts have been made to correlate ER levels with various morphological and other criteria by which breast tumours are classified, but these have produced variable results (for recent review, see Seibert & Lippman, 1982). There may be several reasons why this should be. Most importantly, it is clear that the disease, as judged by hormone receptor content, takes several forms. Most obvious is the well documented recognition of at least four phenotypes for soluble (previously termed 'cytoplasmic', Walters, 1985) receptor status, i.e., ER positive/PR positive, ER positive/PR negative, ER negative/PR positive, ER negative/PR negative. Additional variants are recognised when nuclear-bound receptors are also considered. Further evidence for heterogeneity also emerges when receptor affinities are taken into account (Puddefoot *et al.*, 1986). Moreover, data accumulated in the last few years has shown quite clearly that such heterogeneity is even to be found in the tumour tissue of individual patients. For example, serial samples from an individual tumour may show extremely different

**Figure 3a–i** Progesterone receptor (PR) content for the same samples as in **Figure 2**. Key also as in **Figure 2**. Values for PR content are expressed as fmol mg$^{-1}$ protein. The PR distribution is not closely related to ER (cf. **Figure 2**). In particular, the tumour is not always relatively rich in PR compared with the remainder of the breast.

receptor contents (Tilley *et al.*, 1978; Braunsberg, 1975; Silfersward *et al.*, 1980; van Netten *et al.*, 1985). Recently, too, the development of monoclonal antibodies to receptors has made possible the application of immunohistochemical techniques which in some cases show adjacent ER positive and ER negative tumour cells (King *et al.*, 1985).

The present results confirm and extend these findings. One striking observation is that patients show extraordinary diversity in the nature of their disease. Not only do nearly all show benign breast disease as well as cancer (Figures 2, 3) but in addition some have multiple tumours. In case 17, all three were ER positive/PR positive, but other cases show multifocal tumours of more than one soluble receptor phenotype. For example, case 8 has both ER positive/PR positive and ER positive/PR negative tumours, while case 4 has an ER positive/PR negative and an ER negative/PR positive tumour. Case 5 combines two distinct ER positive/PR positive tumours with, remarkably, a second phenotype (ER negative/PR negative). This probably means that there is no simple relationship between receptor phenotype and oncogene expression. On the other hand, it is very clear that in ER positive tumours, the receptor concentration is generally vastly in excess of that found in the remainder of the tissue (see Table I). The same relationship is not unerringly found for PR. In some cases PR content of PR positive tumours is higher than in the other tissue (e.g. cases 1 and 2, see Figure 3) although comparison with Figure 2 shows the effect is not so marked as for ER. In other cases (e.g. 10 and 17) highest PR values are found in non-tumour tissue. There would appear to be no relationship between ER status and tumour differentiation (Table I), although it is possible that poorly differentiated tumours have low PR content.

It is obviously difficult to be sure that the material divided between histopathologist and biochemist is always quite homogeneous. In this connection some samples of relatively high receptor content were found in fat or connective tissue, (e.g. cases 1, 10, Figure 2). Here it may be suspected that the samples were not identical, and those taken for receptor analysis may have contained tumour cells in addition to normal tissue.

With this caveat in mind, however, it is nevertheless clear that the data supports the hypothesis that ER concentration in ER positive tumours reflects the transformed function of neoplastic tissue (Israel & Band, 1984). Following other work (Green *et al.*, 1986), it also suggests that the expression of the oncogene involved may be related to ER functions.

J.R.P. and E.A. are generously supported by the Cancer Committee and the Joint Research Board of St. Bartholomew's Hospital.

We are grateful to Professor H.D. Ritchie, Emeritus Professor of the University of London, for his encouragement and support, and Winthrop Laboratories, whose funding of an on-going clinical breast trial made this work possible.

### References

ALLEGRA, J.C., LIPPMAN, M.E., GREEN, L. & 5 others (1979). Estrogen receptor values in patients with benign breast disease. *Cancer*, **44**, 228.

BLOOM, M.J.G. & RICHARDSON, W.W. (1957). Histological grading and prognosis in breast cancer. *Br. J. Cancer*, **11**, 359.

BRAUNSBERG, H. (1979). Factors influencing the estimation of oestrogen receptors in human malignant breast tumours. *Eur. J. Cancer*, **11**, 499.

GREEN, S., WALTER, P., KUMAR, V. & 4 others (1986). Human oestrogen receptor cDNA. Sequence, expression and homology to v-erb-A. *Nature*, **320**, 134.

HUBAY, C.A., ARAFAH, B., GORDON, N.H., GUYTON, S.A. & CROWE, J.P. (1984). Hormone receptors: An update and application. *Surg. Clin. North Am.*, **64**, 1155.

ISRAEL, L. & BAND, P. (1984). Hormones as cancer growth factors. *Lancet*, **i**, 843.

JACQUEMIER, J.D., ROLLAND, P.H., VAGUE, D., LIEUTAND, R., SPITALIER, J.M. & MARTIN, P.M. (1982). Relationships between steriod receptor and epithelial cell proliferation in benign fibrocystic disease of the breast. *Cancer*, **49**, 2534.

JOHANSSON, H., TERENIUS, L. & THOREN, L. (1970). The binding of estradiol-17B to human breast cancer and other tissues *in vitro*. *Cancer Res.*, **30**, 692.

KING, R.J.B., REDGRAVE, S., HAYWARD, J.L., MILLIS, R.R. & RUBENS, R.D. (1979). The measurement of receptors for oestradiol and progesterone in human breast tumours. In *Steroid receptor assays in human breast tumours: Methodological and clinical aspects*, King, R.J.B. (ed). p. 55. Alpha-Omega Publishing: Cardiff.

KING, W.J., DESOMBRE, E.R., JENSEN, E.V. & GREENE, G.L. (1985). Comparison of immunocytochemical and steroid binding assays for oestrogen receptor in human breast tumours. *Cancer Res.*, **45**, 293.

LEIGHT, G.S., WELL, S.A. & MCCARTY, JR. K.S. (1984). Sex steroid receptor concentration in breast carcinoma tissue: Effect of devascularization during mastectomy. *Surgery*, **95**, 256.

LOWRY, O.H., ROSEBURGH, N.J., FARR, A.L. & RANDALL, R.J. (1951). Protein measurement with the Folin-phenol reagent. *J. Biol. Chem.*, **193**, 265.

PUDDEFOOT, J.R., ANDERSON, E., VINSON, G.P. & GILMORE, O.J.A. (1986). Heterogeneity of oestrogen receptors in human breast tumours. *J. Endocrinol.*, **108** (suppl.), 105.

SEIBERT, K. & LIPPMAN, M. (1982). Hormone receptors in breast cancer. *Clin. Oncol.*, **1**, 735.

SILFERSWARD, C., SKOOG, L., HUMLA, S., GUSTAFSSON, S.A. & NORDENKSJOLD, B. (1980). Intratumoral variation of cytoplasmic and nuclear estrogen receptor concentrations in human mammary carcinoma. *Eur. J. Cancer*, **16**, 59.

TEICHNER, I., TINKER, M.A., AUGUSTE, L.J., LAUFER, H., STEIN, T.A. & 1 other (1985). Effect of operative devascularization on estrogen and progesterone receptor levels in breast cancer specimens. *Surgery*, **98**, 784.

TERENIUS, L., JOHANSSON, H., RIMSTEN, A. & THOREN, L. (1974). Malignant and benign human mammary disease: Oestrogen binding in relation to clinical data. *Cancer*, **33**, 1364.

TILLEY, W.D., KEIGHTLEY, D.D. & CANT, E.L. (1978). Intersite variation of oestrogen receptors in human breast cancers. *Br. J. Cancer*, **43**, 59.

VAN NETTEN, J.P., ALGARD, F.T., COY, P. & 6 others (1985). Heterogeneous estrogen receptor levels detected via microsamples from individual breast cancers. *Cancer*, **56**, 2019.

WALTERS, M.R. (1985). Steroid hormone receptors and the nucleus. *Endocrine Rev.*, **6**, 512.